# The first Canadian experience with the Afirma® gene expression classifier test

**DOI:** 10.1186/s40463-017-0201-7

**Published:** 2017-04-04

**Authors:** Emily Kay-Rivest, Jamie Tibbo, Sarah Bouhabel, Michael Tamilia, Rebecca Leboeuf, Veronique-Isabelle Forest, Michael P. Hier, Loren Savoury, Richard J. Payne

**Affiliations:** 1 Department of Otolaryngology - Head and Neck Surgery, McGill University, Jewish General Hospital, 3755 Côte-Sainte-Catherine Road, Montréal, QC H3T 1E2 Canada; 2 Department of Otolaryngology - Head and Neck Surgery, Memorial University, St. Clare’s Mercy Hospital, St. John’s, NF Canada; 3 Department of Internal Medicine, Division of Endocrinology, McGill University, Jewish General Hospital, Montreal, QC Canada; 4 Department of Internal Medicine, Division of Endocrinology, University of Montreal, Montreal, QC Canada

**Keywords:** Indeterminate thyroid nodules, Molecular testing, Thyroid cancer, Afirma® Gene Expression Classifier, Ultrasound-guided fine-needle aspiration

## Abstract

**Background:**

Thyroid nodules are common and often benign, although prove to be malignant upon surgical pathology in 5–15% of cases. When assessed with ultrasound-guided fine-needle aspiration (USFNA), 15–30% of the nodules yield an indeterminate result. The Afirma® gene expression classifier (AGEC) was developed to improve management of indeterminate thyroid nodules (ITNs) by classifying them as “benign” or “suspicious.” Objectives were (1) to assess the performance of the AGEC in two Canadian academic medical centres (2), to search for inter-institutional variation and (3) to compare AGEC performance in Canadian versus American institutions.

**Methods:**

We undertook a retrospective cohort study of patients with indeterminate cytopathology (Bethesda Class III or IV) as per USFNA who underwent AGEC testing. We reviewed patient demographics, cytopathological results, AGEC data and, if the patient underwent surgery, results from their final pathology.

**Results:**

In total, we included 172 patients with Bethesda Class III or IV thyroid nodules underwent AGEC testing, 109 in Montreal, Quebec and 63 in St. John’s, Newfoundland, in this study. Among the nodules sent for testing, 55% (60/109) in Montreal and 46% (29/63) in St. John’s returned as “benign.” None of these patients underwent surgery. On the other hand, 45% (49/109) nodules in Montreal and 54% (34/63) in St. John’s were found to be “suspicious,” for a total of 83 specimens. Seventy seven of these patients underwent surgery. Both in Montreal and St. John’s, the final pathology yielded malignant thyroid disease in approximately 50% of the specimens categorized as “suspicious.” Since 2013, no patient diagnosed with a benign nodule as per AGEC testing was found to harbor a malignant thyroid nodule on follow-up.

**Conclusions:**

Molecular analysis is increasingly used in the management of indeterminate thyroid nodules. This study highlights the experience of two Canadian centres with AGEC testing. We found inter-institutional variability in the rate of nodules returning as “benign,” however we found similar rates of confirmed malignancy in nodules returning as “suspicious.” According the literature, results for AGEC testing in two Canadian institutions align with results reported in American centres.

## Background

In the past three decades, an important rise in the incidence of thyroid cancer has been noted worldwide [1]. Currently, ultrasound-guided fine-needle aspiration (USFNA) is the diagnostic tool of choice for evaluation and management of thyroid nodules [2, 3]. However, in approximately 15–30% of USFNAs, the cytological results are found to be indeterminate [4]. Indeterminate thyroid nodules (ITN) include, as per the Bethesda System for Reporting Thyroid Cytopathology (BSRTC), nodules that are Bethesda Class III: atypia of undetermined significance or follicular lesion of undetermined significance (AUS/FLUS) or Bethesda Class IV: follicular neoplasm (FN). A large number of patients with ITNs subsequently undergo diagnostic hemi-thyroidectomy. Studies have shown an overall postoperative malignancy rate ranging between 15 and 30% [5].

Molecular analysis of USFNA specimens from ITNs has gained in popularity recently as an adjunct to microscopic cytological evaluation. Many molecular markers have shown promise, such as BRAF and RAS mutations. Although these markers demonstrate a high specificity and positive predictive value, they have limited sensitivity [6]. A test with a poor sensitivity cannot rule out a diagnosis with certainty. Thus, Chudova et al. set out to find genetic markers with a higher sensitivity in order to develop a test that would confirm benignity of an ITN with greater fidelity [4].

The Afirma® gene expression classifier (AGEC) is a diagnostic test that measures the expression of 167 gene transcripts in specimens of thyroid tissue collected by fine-needle aspiration. It classifies the aspirate as either “benign” or “suspicious.” In a validation study by Alexander et al., the AGEC test was shown to have a sensitivity exceeding 90% and a negative predictive value (NPV) of 94% for both Bethesda Class III and IV nodules [6]. Our study aimed to evaluate the experience with the AGEC test in two Canadian centres: Montreal, Quebec and St. John’s, Newfoundland. To our knowledge, there are no reports in the literature describing the experience of a Canadian centre that uses the AGEC test. We sought to assess the performance of the AGEC test in both centres, to search for inter-institutional variability, and to compare our results with data published from studies in the United States.

## Methods

### Study design

The principal investigators undertook a retrospective cohort study of patients with cytologically indeterminate thyroid nodules who underwent AGEC testing between 2013 and 2015 in two Canadian medical centres: in Montreal, Quebec and in St. John’s, Newfoundland. Multi-center ethics review approval was obtained from Research Ethics Committees at the Jewish General Hospital in Montreal, Québec and at St. Clare’s Mercy Hospital in St. John’s, Newfoundland.

### Patient selection

Patients included were over the age of 18, with one or more thyroid nodules exceeding 1 cm in diameter on ultrasound. The patients included were those with indeterminate cytopathology (Bethesda Class III or IV) as per USFNA. In Montreal, the cytopathology was performed using cell blocks and a Bethesda score was assigned by the pathologist. All USFNA cytology was read internally by experienced thyroid nodule cytopathologists. Upon receipt of indeterminate cytopathology, patients were counseled as to the different management options (surgery, close follow up, and AGEC testing). Patients were counseled at length as to the benefits and pitfalls of each option. Excluded patients were those awaiting surgery or awaiting final pathology results at the time of our data collection.

### Sample collection

Upon obtaining patient consent, a trained physician performed the USFNA to collect the samples for AGEC testing. Proper preservation and packing of the samples was ensured. Each sample was sent via courier to Veracyte’s Clinical Laboratory Improvement Amendments in San Francisco, California. Communication was maintained with customs throughout the process, and receipt confirmation was obtained upon arrival of the specimens.

### Data collection

For each patient the following was reviewed: patient demographics, cytopathological results, AGEC data and, if the patient underwent surgery, results from their final pathology.

### Statistical analysis

Statistical analysis was performed using MedCalc version 12.2. When appropriate, the chi-square test was used. *P-*values less than 0.05 were considered statistically significant.

Also, we calculated AGEC-benign call rate, representing the number AGEC tests returning as benign, and positive predictive value (PPV), representing true positives divided by the number of GEC-suspicious results and compared these to values obtained in the study by Alexander et al. to search for inter-institutional variability.

## Results

In total, 172 patients with Bethesda Class III or IV thyroid nodules underwent AGEC testing were included in this study, 109 in Montreal and 63 in St. John’s. Table 1 shows the distribution of nodules, according to Bethesda classification. In Table 2, we find the AGEC results by institution. In Montreal, 55% (60/109) of nodules returned as “benign.” In St. John’s, 46% (29/63) of nodules returned as “benign.” In St. John’s, ten nodules were returned initially as “no result.” Following this, in five patients, AGEC was repeated. Three yielded results, however two yielded “no results” a second time. Four patients opted not to repeat AGEC but simply to undergo management based on repeat USFNA. Finally, one patient was lost to follow up and was excluded from the study. Only the patients who had a “benign” or “suspicious” result were included in our statistical analysis, however we thought it was important to include these results. In Montreal, this occurred in only one instance; the specimen was collected again and a result obtained.

**Table 1:** Thyroid nodule Bethesda classification in both centres

Nodule Class	B3	B4	Total
Montreal	84 (77%)	25 (23%)	109
St. John’s	21 (33%)	42 (67%)	63
			172

**Table 2:** Afirma® GEC results, by centre

	GEC benign	GEC suspicious	GEC no result	Total
Montreal	60 (55%)	49 (45%)	0^a^	109
St. John’s	29 (41%)	34 (49%)	7^a^ (10%)	70
				179

^a^See further explanation in result section. AGEC tests yielding no results were excluded from our statistical analysis

Overall, 83 out of 172 specimens were classified as “suspicious” according to the AGEC test. Seventy seven patients with “suspicious” nodules had undergone surgery at the time of our data collection, with six patients having decided to not undergo surgery despite the suspicious result. Among the 77 patients who underwent surgery, the ratio of benign to malignant final pathology is nearly 50%. More precisely, in Montreal, 48% (21/44) of “suspicious” nodules were malignant on final pathology, and 52% (17/33) in St. John’s. The summary of these results can be found in Tables 3.

**Table 3:** Final pathology results of patients with “suspicious” Afirma® GEC results

	Underwent surgery	Benign	Malignant
Montreal	44	23 (52%)	21 (48%)
St. John’s	33	16 (48%)	17 (52%)
Total	77	39 (51%)	38 (49%)

Finally, we evaluated pre-test probability (or disease prevalence) and the post-test probability of a positive AGEC test. The disease prevalence of malignancy among all Bethesda 3 and 4’s (among all patients, not only this particular group of patients who elected or were chosen to undergo AGEC testing) was 32% in Montreal and 46% in St. John’s. The post-test probability of a positive test (which is the PPV) was 45.71% [95% confidence interval (CI) 29.22–63.13] in Montreal and 51.51% [95% CI 33.85–68.83] in St. John’s. Finally, since the start of the study, no patients with benign results as per AGEC testing were found to harbor a malignant thyroid nodule on follow-up.

## Discussion

Interest in the combination of microscopic thyroid cytopathology and molecular analysis has increased significantly in recent years. Molecular analysis has gained popularity with the discovery that up to two-thirds of follicular cell-derived thyroid malignancies harbor a genetic mutation [7–9]. The AGEC test is used to reclassify cytologically indeterminate thyroid nodules, categorizing the nodules as either “benign” or “suspicious” based on the expression pattern of mRNA extracted from the USFNA samples. The test uses an algorithm comprised of 167 genes. 142 of them are part of the main classifier to separate benign versus malignant disease; whereas the other 25 genes are used to filter out rare neoplasms. [6] The test is meant to predict benignity and avoid unnecessary diagnostic surgery and is reported to have a high sensitivity as well as a high negative predictive value. Our study evaluated the experience of two Canadian centres using the AGEC test. To date, studies of the AGEC test have included two large clinical validation studies [4, 6], three studies evaluating clinical utility [10–12] one analytical validation study [13], as well as a quality of life and cost-effectiveness study [14]. In addition, there have been over a dozen single institution reports recounting experiences with the test. [15–20] However, these studies were performed by investigators in the United States. To our knowledge, there are no reports published describing the use of this test in Canada.

We investigated 172 patients with indeterminate thyroid nodule cytology who underwent Afirma® testing. In total, 89 nodules-60 (55%) in Montreal and 29 (46%) in St. John’s-returned as “benign” as per AGEC testing. This result significantly impacted patient management, as no patient underwent surgery. Traditionally, the vast majority of these patients would have undergone a diagnostic hemi-thyroidectomy at these centres. Instead, these patients avoided surgery and are currently managed with regular ultrasound follow-up. Unfortunately, the long-term outcome for these patients is still unknown.

Additionally, 45% of patients in Montreal and 54% of patients in St. John’s had “suspicious” results as per the AGEC test. In Montreal, 48% of “suspicious” nodules were malignant on final pathology, and 52% in St. John’s. In reality, receiving a “suspicious” AGEC test in our Canadian cohort conferred a risk of malignancy of approximately 50%, which is greater than the 5–15% (Bethesda 3) or the 15–30% (Bethesda 4) suggested in the Bethesda classification. We must keep in mind that this does not indicate that 50% of B3/B4’s in our centres are malignant, because many were removed from this part of the analysis after having been classified as “benign” by AGEC. The final pathology outcomes of “suspicious” nodules were similar in St. John’s and Montreal. Indeed, in the above-mentioned study by Alexander et al., the rates of malignant final pathology varied greatly between sites, with malignancy rates as low as 33% and as high as 80% at certain centres. However, multiple single-institution studies, such as that of Lastra et al. and Marti et al., have shown approximately 50% malignancy rates in “suspicious” nodules as per AGEC, mirroring our study [15, 18]. As most patients with a “suspicious” nodule undergo surgery, it implies that AGEC testing in these cases did not necessarily impact management, since traditionally most of these patients would have undergone diagnostic surgery. Indeed, when we compared patients from our own cohort with ITNs with “suspicious” results as per AGEC testing compared to those in whom AGEC testing was not performed, the final surgical pathology outcomes were nearly identical.

There is a 9% difference in rates of “benign” nodules as per AGEC between institutions. Data published by Veracyte stated that approximately 50% of samples submitted are reported as benign. A slightly higher rate was found in Montreal (55% of specimens returned as “benign”), and a slightly lower number (46%) of benign results were obtained in St. John’s. This discrepancy in benign rates is found throughout the literature. For example, a community-based practice study by Harell et al. reported a 34% benign rate, and a study by McIver et al., performed in two academic centres, reported that only 27% of samples returned as “benign” [19, 20]. Another study, published by Alexander et al. in 2013, assessed 339 FNA samples collected from five different academic medical centres. Nearly all patients enrolled were either a Bethesda Class 3 or 4, with eight patients having FNA cytology “suspicious for malignancy”. In this large study, significant variability of “benign” AGEC results were noted between sites, ranging between 38 and 71% [12]. In our study, the variability could in theory be attributed to difference in patient cohorts. For example, St. John’s had more patients with Bethesda Class IV nodules compared to the patients in Montreal. It is also possible that the prevalence of thyroid cancer was greater in the patient population studied in St. John’s.

Finally, in the pivotal validation study published in 2012 by Alexander et al., the AGEC test was found to have a sensitivity of 90%, a specificity of 53% and a NPV of 95% [6]. In our study, due to lack of surgical follow-up among “benign,” we cannot assess a true NPV. The high NPV achieved by Alexander et al. cannot necessarily be replicated in all clinical scenarios, as NPV is dependent on prevalence of disease in a given population.

### Limitations

Our study has several limitations. First is the lack of surgical follow-up for patients with “benign” AGEC results. Although these patients are followed with regular ultrasound examinations in both centres, it will take several years before we know the true nature of these thyroid nodules. Another limitation in our study is a possible lack of concordance between cytological reporting at our centres. We found slightly different rates of benign and malignant results, and suspect discordance in pathology and prevalence of malignancy among the populations studied. Finally we make a note regarding the rate of “no result” specimens. The team in St. John’s discussed this issue with the company and in the end, it could not be explained. Various needle sizes, including 27 gauge, 25 gauge and 22 gauge needles were tried to mitigate this problem, both with and without aspiration, and did not seem to have an impact on outcomes.

## Conclusion

The results from our study demonstrate that AGEC testing may be a useful adjunct to microscopic cytological testing when evaluating indeterminate thyroid nodules. We found variability between the benign rates in both sites, but the overall results are comparable to previously published studies. Further long-term follow-up of these patients is needed in order to confirm the usefulness and safety of this test in Canada, however, to date, the test appears safe and in our cohort, no patients have with a “benign” nodule as per AGEC have presented with malignant thyroid disease. For now, AGEC testing shows promise, but neither cytological nor genetic testing alone should direct management of a patient with a thyroid nodule.

## Abbreviations

USFNA: Ultrasound-guided fine-needle aspiration
FNA: Fine-needle aspiration
AGEC: Afirma® gene expression classifier
ITN: Indeterminate thyroid nodules
BSRTC: Bethesda System for Reporting Thyroid Cytopathology
AUS/FLUS: atypia of undetermined significance or follicular lesion of undetermined significance
FN: Follicular neoplasm
PPV: Positive predictive value
NPV: Negative predictive value
